# The effect of flexor tenotomy on healing and prevention of neuropathic diabetic foot ulcers on the distal end of the toe

**DOI:** 10.1186/1757-1146-6-3

**Published:** 2013-01-24

**Authors:** Jaap J van Netten, Adriaan Bril, Jeff G van Baal

**Affiliations:** 1 Department of surgery, Hospital Group Twente, Almelo, PO Box 7600, Almelo, SZ, 7600, the Netherlands

**Keywords:** Flexor tenotomy, Diabetic foot, Diabetic neuropathy, Wound healing

## Abstract

**Background:**

Flexor tenotomy is a minimally invasive surgical alternative for the treatment of neuropathic diabetic foot ulcers on the distal end of the toe. The influence of infection on healing and time to heal after flexor tenotomy is unknown. Flexor tenotomy can also be used as a prophylactic treatment. The effectiveness as a prophylactic treatment has not been described before.

**Methods:**

A retrospective study was performed with the inclusion of all consecutive flexor tenotomies from one hospital between January 2005 and December 2011.

**Results:**

From 38 ulcers, 35 healed (92%), with a mean time to heal of 22 ± 26 days. The longest duration for healing was found for infected ulcers that were penetrating to bone (35 days; p = .042). Cases of prophylactic flexor tenotomies (n=9) did not result in any ulcer or other complications during follow-up.

**Conclusions:**

The results of this study suggest that flexor tenotomy may be beneficial for neuropathic diabetic foot ulcers on the distal end of the toe, with a high healing percentage and a short mean time to heal. Infected ulcers that penetrated to bone took a significantly longer time to heal. Prospective research, to confirm the results of this retrospective study, should be performed.

## Background

Foot ulcers are a frequently occurring and costly complication of diabetes, with a yearly incidence around 2% [1]. Claw and hammer toe deformities frequently develop in people with diabetes, leading to increased pressure on the distal end of the toes [2]. In combination with neuropathy, this may lead to abundant callus and the development of ulcers. Conservative treatment of these ulcers consists of wound care, sharp debridement and off-loading of the foot by means of shoe adaptations or casting [3]. A minimally invasive surgical alternative is flexor tenotomy [4-9].

Four small retrospective studies have recently been published, describing positive results of flexor tenotomy: healing rates of 98% to 100% [6-9] after flexor tenotomy, and mean time to heal ranging from 21 to 56 days [6-9]. However, limited patient information was provided in those studies, leaving relevant questions unanswered [4]. For example, the influence on healing and time to heal of preoperative treatment, ulcer duration before flexor tenotomy, ulcer location or infection at the moment of flexor tenotomy are all unknown.

Flexor tenotomy can also be performed for prevention of diabetic foot ulcers, when abundant callus is present on the distal end of claw and hammer toes. It was described in one study that in ten cases the flexor tenotomy was prophylactic [7]. Unfortunately no further details were provided regarding effectiveness or complications during follow-up of this prophylactic surgery [7]. The preventative effects of flexor tenotomy are still unknown.

The aim of this study was to retrospectively investigate all consecutive flexor tenotomies in people with neuropathic diabetic foot ulcers on the distal end of the toe, to report healing and time to heal, and to investigate the influence of preoperative treatment, ulcer duration before flexor tenotomy, ulcer location, and infection on healing and time to heal. The second aim was to describe the outcomes of all consecutive prophylactic flexor tenotomies in patients with diabetes and claw or hammer toe deformities.

## Methods

### Study design

A retrospective cohort study was performed including all consecutive flexor tenotomies in patients with diabetes mellitus, performed in one hospital and by one surgeon between January 2005 and December 2011. For this retrospective study, no ethical approval was required. A database containing all surgical procedures in the study period was scanned for the code representing flexor tenotomy, after which a list was compiled of all patients who had undergone a flexor tenotomy.

The medical files of these patients were reviewed for: (i) patient characteristics (gender, date of birth, diabetes type, neuropathy assessed with a 10 g Semmes-Weinstein monofilament, peripheral arterial disease assessed by palpation of pulses in the lower limb followed by hand-held Doppler evaluation of the flow signals from both foot arteries and measurement of toe pressure, pre-operative treatment); (ii) ulcer characteristics (duration before flexor tenotomy, location, presence of infection based on clinical assessment, depth divided in three groups as superficial, penetrating to tendon or capsule, penetrating to bone or joint, University of Texas Wound Classification grade and stage [10], based on presence of peripheral arterial disease, infection and depth, date of flexor tenotomy, date of ulcer healing, complications amputation re-ulceration (development of a new ulcer on the same location, after the previous ulcer was healed) and shifted flexor tenotomy (when a subsequent flexor tenotomy was performed on the same foot but on a different toe, the subsequent flexor tenotomy was listed as shifted flexor tenotomy) during follow-up).

### Flexor tenotomy

All flexor tenotomies in the study period were performed by one surgeon, following a standard protocol [7,8]. Patients underwent outpatient flexor tenotomy under a selective block using 2% lidocaine without adrenaline. The flexor digitorum longus is placed under tension (‘bow-stringing’) by positioning the ankle joint in dorsiflexion, while at the same time positioning the affected toe in hyperextension. A stab-wound incision (around 3 mm) is made at the middle of the proximal phalanx, and the tendon is cut. All stab wounds are sutured and a pressure bandage is applied for the first days. The foot remains offloaded for 24 hours, after which the patient can weightbear again. The patient is examined at 1-week, and subsequently followed-up at regular intervals.

### Data analysis

Data were analyzed using SPSS for Windows, version 17.0 (SPSS Inc., Chicago IL, United States of America). Differences between groups were tested using non-parametric tests, because of non-normal distribution of the data.

## Results

A total of 47 flexor tenotomies were performed on 33 patients in the study period. Of these flexor tenotomies, 38 were performed because of an ulcer and 9 were prophylactic. Patient, foot and ulcer characteristics are described in Tables 1 (group characteristics). For individual characteristics see the Additional file 1.

**Table 1:** Characteristics of all patients

**Patient characteristics (n=33)**		
Gender	M:	17
	F:	16
Age (years)	Mean±SD	69 ± 12
	(range)	(41 – 93)
Diabetes type	Type 1:	0
	Type 2:	33
Neuropathy*	Yes:	31
	No:	1
	Unknown:	1
PAD	Yes:	0
	No:	33
Foot characteristics (n=47)		
Pre-operative Treatment	Orthopedic shoe:	14
	Cast shoe:	3
	Orthosis:	5
	Felt:	2
	None:	23
Location ^†^	Digit 1:	15
	Digit 2:	16
	Digit 3:	15
	Digit 4:	1
Ulcer characteristics (n=38)		
Ulcer duration (days)	Mean±SD	96 ± 112
	(range)	(9 – 525)
Ulcer classification ^‡^	1A:	14
	1B:	6
	3B:	18

Note: Values are n, or as indicated; SD = standard deviation; PAD = peripheral arterial disease, scored as no when foot arteries were palpable by the surgeon; *: neuropathy was unknown for 1 patient, 1 patient had no neuropathy and no ulcer (prophylactic flexor tenotomy); ^†^: no difference between left and right is made. ^‡^: ulcer classification = University of Texas Wound Classification system; [10]; Cast shoe = MAnning-Baal-ALmelo cast shoe; [11].

Of the 38 ulcers, 35 healed (92%) with a mean time to heal of 22 ± 26 days. No relation was found between healing and ulcer duration before flexor tenotomy, pre-operative treatment, ulcer location or ulcer classification (Table 2), although the three ulcers that did not heal were all infected and penetrated to bone (Table 2). A significant relation was found between time to heal and ulcer classification, with the shortest time to heal in superficial ulcers without infection, and the longest time to heal seen in infected ulcers penetrating to bone (13 vs. 35 days; p = .042; Table 3). No relation was found between time to heal and ulcer duration before flexor tenotomy, pre-operative treatment or ulcer location (Table 3).

**Table 2:** Relation between healing after flexor tenotomy and ulcer characteristics

		**Not healed (n=3)**	**Healed (n=35)**	**p-value**
Ulcer duration* (days)	Mean±SD	131±175	93±108	.828 ^‡^
	(range)	(23–333)	(9–525)	
Pre-operative treatment	Orthopedic shoe:	1	9	.711 ^§^
	Cast:	1	2	
	Orthosis:	0	4	
	Felt:	0	2	
	None	1	18	
Ulcer location ^†^	Digit 1:	2	10	.178 ^§^
	Digit 2:	1	14	
	Digit 3:	0	11	
Ulcer classification ^‡^	1A:	0	14	.259 ^§^
	1B:	0	6	
	3B:	3	15	

Note: Values are n or as indicated; OS = orthopaedic shoes; Cast shoe = MAnning-Baal-ALmelo cast shoe; [11]; sign. = significance; *: duration unknown for three healed ulcers; ^†^: no difference between left and right is made; ^‡^: ulcer classification = University of Texas Wound Classification system; [10]; ^‡^: Mann–Whitney U test; ^§^: Fisher’s Exact test.

**Table 3:** Relation between time to heal after flexor tenotomy and ulcer characteristics

		**Time to heal (days) (n=35)**	**Sign**
Ulcer duration*	Correlation ^§^	.170	.329 ^§^
Pre-operative treatment	Orthopedic shoes:	15 ± 10	.467 ^†^
	Cast:	24 ± 17	
	Orthosis:	21 ± 3	
	Felt:	10 ± 4	
	None	28 ± 34	
Ulcer location ^†^	Digit 1:	15 ± 6	.483 ^ζ^
	Digit 2:	24 ± 17	
	Digit 3:	27 ± 43	
Ulcer classification ^‡^	1A:	13 ± 6	.042 ^ζ^
	1B:	18 ± 9	
	3B:	33 ± 37	

Note: Values are mean ± standard deviation; Cast = MAnning-Baal-ALmelo cast shoe;(11) sign. = significance; *: duration unknown for three ulcers; ^†^: no difference between left and right is made; ^‡^ : ulcer classification = University of Texas Wound Classification system;(10) ^§^: Spearman’s ρ; ^ζ^: Kruskal-Wallis test.

Mean follow-up time was 23 ± 11 months (range: 11 – 60 months). Complications of amputation (n=3) and re-ulceration (n=7) were only found in infected ulcers penetrating to bone at the moment of flexor tenotomy (see Additional file 1). Toe amputation was performed for all three cases with an ulcer that did not heal, after 29, 68, and 134 days. Reason for amputation was spreading of the infection. All amputation wounds healed. Re-ulceration occurred on the plantar side of the toe (n=6), and on the dorsal side of the toe (n=1). The latter was the result from dorsiflexion of the metatarsophalangeal joint caused by the extensor digitorum longus muscle, even though there was no active muscle contraction. Treatment after re-ulceration consisted of antibiotics and shoe adaptations. Eight tenotomies were listed as shifted, meaning they were performed on the same foot after the first flexor tenotomy (see Additional file 1).

Nine flexor tenotomies were prophylactic, performed when abundant callus was present on the distal end of the toe (see Additional file 1). None of these toes developed an ulcer or any other complication during follow-up (see Additional file 1).

## Discussion

Flexor tenotomy is a minimally invasive surgical procedure for off-loading claw and hammer toe deformities. In this retrospective study, previously reported findings that diabetic foot ulcers on the distal end of the toe heal quickly after flexor tenotomy were replicated [6-9]. The rate of non-healing ulcers found in the population of this study is slightly higher (9%) than seen in recently published retrospective studies (0% - 2% [6-9]). All three ulcers that did not heal were infected and penetrated to bone at the moment of flexor tenotomy. Re-ulceration was a complication found in seven patients, which is slightly higher than previous studies where this was described [6,8]. As with amputation, re-ulceration occurred only in toes that were infected and penetrating to bone at the moment of flexor tenotomy. With scarce information on infection available from previous studies, options to reliably compare these findings are unfortunately limited. Infected ulcers penetrating to bone are not a contra-indication for performing flexor tenotomy, as the majority of these ulcers healed and approximately half of them without complications. However, with amputation and re-ulceration only found in this group, the timing of the flexor tenotomy should be investigated in respect to performing the procedure earlier or later (before an ulcer becomes infected and penetrating to bone is possible, or when an infection is cleared and penetrating to bone is no longer possible), or if a different treatment (e.g. off-loading by means of cast or shoe adaptations) is preferred.

A complication that has been described before is shifting of the foot problem [6]. After successful flexor tenotomy, the next toe may develop an ulcer due to the increased pressure under that toe. The change to the structure and therefore function of the diabetic foot has implications for a shift in plantar pressures and other forces affecting the foot, sometimes resulting in a new ulcer on the next toe. Shifted flexor tenotomy is then one of the first treatment options to consider. With eight shifted flexor tenotomies found in our population, this is not a rare complication. Frequent follow-up visits are essential to timely detect these ulcers, or the abundant callus which is a pre-sign of these ulcers. A complication after flexor tenotomy that has not been described before was dorsiflexion of the metatarsophalangeal joint. This may be caused by the extensor digitorum longus muscle, without active muscle contraction, or by the inadvertent release of the flexor brevis muscle. This was found in one patient, one month after the flexor tenotomy. There was inadequate space in the toe-box of the shoe due to the altered toe position, and consequently an ulcer developed on the dorsal side of the toe. Frequent follow-up visits and timely made shoe adaptations can prevent ulceration due to this complication.

Prophylactic flexor tenotomies have only been described once, yet without describing the effectiveness of the prophylactic intervention [7]. In this study, prophylactic flexor tenotomy was successful in the prevention of ulcer development during long-term follow-up. This suggests that flexor tenotomy may hold promise as a measure for prevention and might be considered, if possible, in an early stage of treatment, before an ulcer can develop. It should be noted that our sample was limited to nine people only, so more research is needed before we can draw definitive conclusions.

The retrospective nature of this study presents an important limitation. Although prospective studies are preferred [4], this retrospective design was chosen to investigate the relation between ulcer duration, preoperative treatment, ulcer location, or infection on healing and time to heal after flexor tenotomy. The findings, especially concerning the influence of infection and penetrating to bone at the moment of flexor tenotomy, are relevant and useful additions when setting up prospective trials. The effect of flexor tenotomy should be compared with conservative non-surgical treatment options such as off-loading by means of casting or shoe adaptations, and also compared with open surgical procedures such as resection arthroplasty and toe amputation in future research. The influence of infection on negative outcomes seen in this study cohort should be considered further to determine optimal timing for surgical procedures in the diabetic foot.

## Conclusions

The results of this study suggest that flexor tenotomy may be beneficial for neuropathic diabetic foot ulcers on the distal end of the toe, with a high healing percentage and a short mean time to heal. Infection and penetrating to bone should not be considered contra-indications. However, these cases may experience delayed healing times and an increased risk of complications during follow-up. Prophylactic flexor tenotomy may hold promise as a measure for prevention of ulceration on the distal end of the toe. Prospective research, to confirm the results of this retrospective study and to compare flexor tenotomy with other forms of treatment, should be performed before definitive conclusions can be drawn.

## Competing interests

The authors declare that they have no competing interests.

## Authors’ contributions

JvN participated in the design of the study, performed statistical analysis, and drafted the manuscript. AB participated in the design of the study and performed all retrospective data collection. JvB designed the study. All authors made substantial contributions to analysis and interpretation of the data and have been involved in drafting the manuscript. All authors read and approved the final manuscript.

## Supplementary Material

Additional file 1 Characteristics of individual patients. Click here for file
