# Supplementary material for: The effect of flexor tenotomy on healing and prevention of neuropathic diabetic foot ulcers on the distal end of the toe
Source: J Foot Ankle Res. 2013 Jan 24;6:3. doi: 10.1186/1757-1146-6-3 (PMC3565934; doi:10.1186/1757-1146-6-3)
Supplement: Additional file 1 — Characteristics of individual patients. [file 1757-1146-6-3-S1.doc]

Additional file: Characteristics of individual patients

|  | **1** | **2** | **2** | **3** | **4** | **5** | **6** | **6** | **7** | **7** | **7** | **7** | **8** | **9** | **10** | |  |
| --- | --- | --- | --- | --- | --- | --- | --- | --- | --- | --- | --- | --- | --- | --- | --- | --- | --- |
| Gender | F | F |  | F | M | F | F |  | M |  |  |  | F | F | M | |  |
| Age (years) | 85 | 49 |  | 50 | 41 | 58 | 76 |  | 61 |  |  |  | 74 | 87 | 84 | |  |
| Ulcer or proph. | Ulcer | Ulcer | Ulcer | Proph. | Ulcer | Ulcer | Ulcer | Ulcer | Ulcer | Ulcer | Ulcer | Ulcer | Proph. | Ulcer | Ulcer | |  |
| Location | R dig2 | R dig1 | R dig2 | L dig3 | R dig1 | R dig1 | R dig1 | R dig3 | L Dig2 | R dig2 | L dig3 | R dig3 | L dig3 | L dig2 | R dig3 | |  |
| Pre-op. treatm. | None | OS | Orth. | None | Cast | OS | OS | Orth. | None | None | None | None | None | None | None | |  |
| Duration (days) | 160 | 91 | 400 |  | 184 | 81 | 115 | 67 | 48 | 38 | 38 | 17 |  | 92 | 196 | |  |
| Classification | 3b | 1b | 1b |  | 3b | 1b | 1b | 1a | 3b | 1a | 1a | 1b |  | 3b | 1a | |  |
| Surgery date | 09/05 | 05/06 | 07/08 | 06/06 | 09/07 | 02/08 | 03/08 | 07/08 | 06/08 | 08/08 | 08/08 | 10/08 | 08/08 | 09/08 | 12/08 | |  |
| Healed (days) | 57 | 14 | 20 |  | 12 | 29 | 14 | 18 | 8 | 11 | 11 | 4 |  | 26 | 14 | |  |
| Complication* |  |  | shifted |  | re-ulc. |  |  | shifted |  |  | shifted | shifted |  |  |  | |  |
|  | **11** | **11** | **11** | **12** | **13** | **14** | **15** | **15** | **15** | **16** | **17** | **18** | **19** | **20** | **21** | | **22** |
| Gender | M |  |  | F | M | M | M |  |  | M | M | F | F | M | F | | F |
| Age (years) | 63 |  |  | 80 | 68 | 69 | 58 |  |  | 70 | 77 | 65 | 79 | 71 | 87 | | 80 |
| Ulcer or proph. | Ulcer | Ulcer | Proph. | Ulcer | Ulcer | Proph. | Ulcer | Ulcer | Ulcer | Ulcer | Ulcer | Ulcer | Ulcer | Ulcer | Ulcer | | Ulcer |
| Location | L dig1 | L dig3 | L dig4 | L dig2 | R dig1 | R dig1 | L dig2 | L dig1 | R dig2 | L dig1 | L dig2 | R dig3 | L dig2 | R dig1 | R dig1 | | R dig1 |
| Pre-op. treatm. | Cast | OS | OS | OS | OS | None | OS | OS | OS | OS | None | None | Felt | Felt | None | | None |
| Duration (days) | 333 | 35 |  | 100 | 23 |  | 21 | 175 | 21 | ? | 17 | 180 | 26 | 87 | 122 | | 35 |
| Grade | 3b | 1a |  | 1a | 3b |  | 1a | 1a | 3b | 1a | 3b | 3b | 1a | 3b | 1a | | 3b |
| Surgery date | 02/09 | 08/09 | 08/09 | 04/09 | 05/09 | 08/09 | 10/09 | 07/10 | 09/10 | 11/09 | 11/09 | 03/10 | 05/10 | 05/10 | 07/10 | | 10/10 |
| Healed (days) | not | 7 |  | 7 | not |  | 7 | 16 | 33 | 9 | 20 | 28 | 7 | 12 | 14 | | 6 |
| Complication* | amp | shifted | shifted |  | amp |  |  | shifted |  |  | re-ulc. |  |  | re-ulc. |  | | re-ulc. |
|  | **23** | **24** | **25** | **25** | **25** | **26** | **27** | **27** | **28** | **29** | **30** | **31** | **32** | **32** | **33** | **33** | |
| Gender | F | M | F |  |  | M | M |  | F | F | M | M | M |  | M |  | |
| Age (years) | 93 | 68 | 57 |  |  | 65 | 53 |  | 80 | 72 | 60 | 61 | 75 |  | 60 |  | |
| Ulcer or proph. | Ulcer | Ulcer | Proph. | Proph. | Proph. | Ulcer | Ulcer | Ulcer | Ulcer | Ulcer | Ulcer | Ulcer | Ulcer | Proph. | Ulcer | Proph. | |
| Location | L dig3 | L dig2 | R dig1 | R dig2 | L dig1 | L dig2 | L dig2 | L dig3 | L dig3 | R dig3 | L dig3 | R dig2 | L dig1 | R dig3 | L dig2 | L dig3 | |
| Pre-op. treatm. | Orth. | Cast | OS | OS | OS | None | None | None | None | None | None | None | Orth. | Orth. | None | None | |
| Duration (days) | 23 | 13 |  |  |  | 36 | 62 | 19 | 18 | 69 | 35 | 21 | ? |  | 102 |  | |
| Grade | 1b | 3b |  |  |  | 3b | 3b | 3b | 1a | 3b | 3b | 1a | 1a |  | 3b |  | |
| Surgery date | 10/10 | 10/10 | 02/11 | 02/11 | 10/11 | 02/11 | 03/11 | 04/11 | 07/11 | 08/11 | 08/11 | 08/11 | 10/11 | 11/11 | 11/11 | 11/11 | |
| Healed (days) | 26 | 36 |  |  |  | not | 24 | 154 | 7 | 10 | 21 | ? | 21 |  | 110 |  | |
| Complication* |  | re-ulc. |  |  |  | amp | re-ulc. | shifted |  |  |  |  |  |  | re-ulc.^†^ |  | |

Note: Proph = prophylactic flexor tenotomy; Pre-op. treatm. = pre-operative treatment; OS = orthopaedic shoes; Orth. = orthosis; Cast = MAnning-Baal-ALmelo cast shoe;(11) Classification = University of Texas Wound Classification system;(10) na = not applicable; *: the complications amp (=amputation), re-ulc. (=re-ulceration) and shifted (‘shifted flexor tenotomy’) are listed. ^†^: on the dorsal side of the toe.
